# Rift Valley fever virus in small ruminants in the Democratic Republic of the Congo

**DOI:** 10.4102/ojvr.v86i1.1737

**Published:** 2019-10-10

**Authors:** Georges M. Tshilenge, Mfumu L.K Mulumba, Gerald Misinzo, Rob Noad, William G. Dundon

**Affiliations:** 1 Department of Preclinical Medicine, Faculty of Veterinary Medicine, University of Kinshasa, Kinshasa XI, the Democratic Republic of the Congo; 2 Department of Clinical Medicine, Faculty of Veterinary Medicine, University of Kinshasa, Kinshasa XI, the Democratic Republic of the Congo; 3 Department of Microbiology and Parasitology, Sokoine University of Agriculture, Morogoro, United Republic of Tanzania; 4 Department of Pathobiology and Population Sciences, London School of Hygiene and Tropical Medicine, Royal Veterinary College, Hertfordshire, United Kingdom; 5 Animal Production and Health Laboratory, Joint Food and Agriculture Orginazation/International Atomic Energy Agency, Vienna, Austria; 6 Department of Nuclear Sciences and Applications, International Atomic Energy Agency, Vienna, Austria

**Keywords:** Rift Valley fever, ruminants, serology, Democratic Republic of the Congo, prevalence

## Abstract

Rift Valley fever (RVF) is a zoonotic viral disease caused by the RVF phlebovirus (RVFV) that infects a variety of animal species including sheep and goats. Sera (*n* = 893) collected between 2013 and 2015 from randomly selected indigenous sheep and goats in seven provinces of the Democratic Republic of the Congo (DRC) were tested for the presence of specific immunoglobulin G (IgG) and M (IgM) against RVFV, using two commercially available enzyme-linked immunosorbent assays. The reverse transcription polymerase chain reaction (RT-PCR) was also used to detect RVFV nucleic acid. There was significant variation in true seroprevalence of RVFV for both sheep and goats between the seven provinces investigated. Values ranged from 0.0 (95% confidence interval [CI] 0.0–6.55) to 23.81 (95% CI 12.03–41.76) for goat and 0.0 (95% CI 0.0–7.56) to 37.11 (95% CI 15.48–65.94) for sheep, respectively. One serum (1.85%) out of 54 that tested positive for IgG was found to be IgM-positive. This same sample was also positive by RT-PCR indicating an active or recent infection. These findings report the presence of RVFV in small ruminants in the DRC for the first time and indicate variations in exposure to the virus in different parts of the country.

## Introduction

Rift Valley fever (RVF) is a zoonotic viral haemorrhagic fever caused by RVF phlebovirus (RVFV) which is a negative single-stranded ribonucleic acid (RNA) virus that belongs to the order *Bunyavirales*, family *Phenuiviridae* (previously named *Bunyaviridae*) and genus *Phlebovirus* (Plyusnin et al. 2011). The disease infects both large and small ruminants and, as such, has a significant socio-economic impact on livestock in many African countries (Martin et al. 2008). Pregnant ruminants infected with RVFV are subject to high rates of abortion, while high mortality is seen among young animals, characterised by hepatic necrosis and pantropic haemorrhage (Coetzer 1988). The disease was first described in 1931 as an enzootic hepatitis among sheep in Kenya (Daubney, Hudson & Garnham 1931). The spread and dissemination of the disease can be facilitated by a number of different factors including vectors such as mosquitoes, ticks and flies (Linthicum et al. 1985; Mweya et al. 2013; Turell et al. 2007), climate (Anyamba, Linthicum & Tucker 2001; Walsh, Willem de Smalen & Mor 2017) and infected livestock trade and movement (Kenawy, Abdel-Hamid & Beier 2018).

Rift Valley fever phlebovirus is also a significant threat to human health. The virus can be transmitted by mosquito bites, contact with infected tissues, blood or amniotic fluid (especially in aborted material) in slaughterhouses, and handling of contaminated meat during food preparation (Mroz et al. 2017). Prevention and control of RVF in humans therefore rely on preventing the disease in domestic animals in the peridomestic environment. Currently, the best and most efficient way to do this is to vaccinate all susceptible animals (Dungu, Lubisi & Ikegami 2018; Kenawy et al. 2018).

Several countries in Africa, the Arabian Peninsula and islands in the Indian Ocean have reported RVF outbreaks of different severities (King et al. 2010; Madani et al. 2003; Maganga et al. 2017; Nanyingi et al. 2017; Nguku et al. 2010; Oyas et al. 2018; Shoemaker et al. 2002; Sindato et al. 2015). In the Democratic Republic of the Congo (DRC), although no RVF outbreaks have been reported to date, seropositivity of cattle to RVFV was first described in 2009 (Mulumba et al. 2009) and has recently been confirmed (Tshilenge et al. 2018). However, no information is available on the seroprevalence of RVFV in small ruminants (goat and sheep) in the country. This study was therefore undertaken to fill this knowledge gap and to provide a clearer picture of the presence of RVFV in the DRC.

## Materials and methods

**FIGURE 1:**
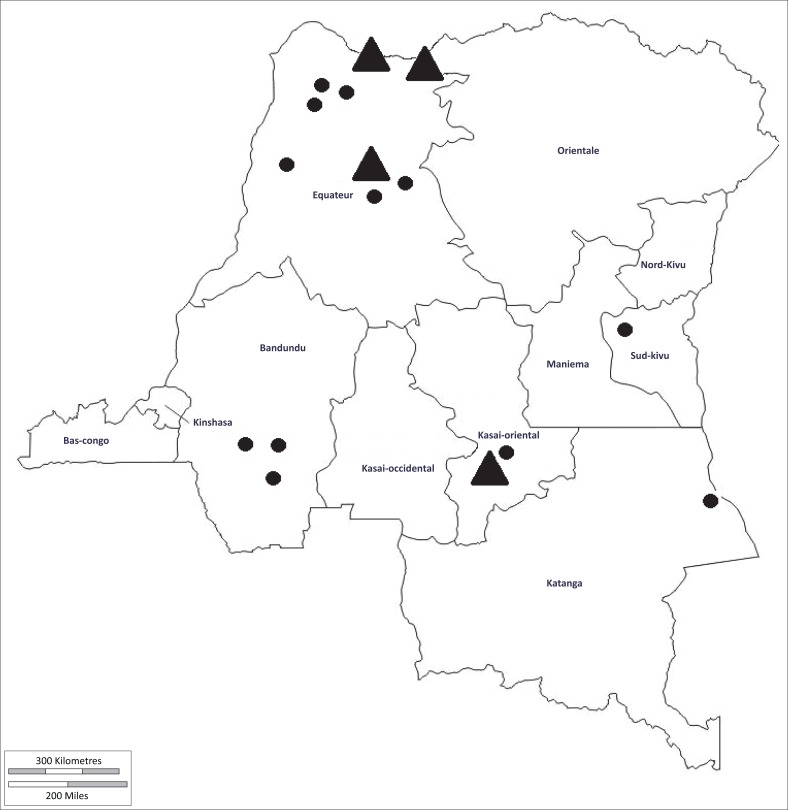
Map of Democratic Republic of the Congo showing locations of sample collection (adapted with permission from www.d-maps.com). Territories with seroprevalences > 10% are indicated with black triangles.

Samples were collected in 7 of the 26 provinces of DRC (Figure 1 and Table 1), namely Mongala (2°09’N, 21°31’E), South Ubangi (3°15’N, 19°46’E), North Ubangi (4°1’N, 21°01’E), Kwilu (5°02’S, 18°50’), Lomami (6°08’S, 24°29’E), South Kivu (2°42’S, -27°2’E) and Tanganyika (7°04’S, 29°43’E).

**TABLE 1:** Serological status by location to anti-Rift Valley fever immunoglobulin G antibodies.

Province	Territories	Caprine				Ovine			
		Tested	Positive	Prevalence	95% CI	Tested	Positive	Prevalence	95% CI
Equateur or *Sud-Ubangi*	Budjala	50	0	0.00	0.0–7.28	4	0	0.00	0.0–49.99
	Gemena	60	4	6.80	2.6–16.25	7	1	14.58	0.75–52.36
	Kungu	56	0	0.00	0.0–6.55	9	0	0.00	0.0–30.53
Equateur or *Nord-Ubangi*	Businga	30	7	23.81	12.03–41.76	11	4	37.11	15.48–65.94
	Bosobolo	10	0	0.00	0.0–28.32	31	1	3.29	0.02–16.52
	Mobayimbongo	22	4	18.55	7.46–39.30	17	5	30.01	13.55–54.22
	Yakoma	23	3	13.31	4.63–32.78	30	4	13.61	5.42–30.29
Equateur or *Mongala*	Bumba	80	3	3.83	1.31–10.67	48	0	0.00	0.0–7.56
	Lisala	62	1	1.65	0.03–8.76	34	1	3.00	0.02–15.22
Bandundu or *Kwilu*	Idiofa	17	0	0.00	0.0–18.81	8	0	0.00	0.0–33.10
	Masi-Manimba	47	2	4.34	1.20–14.54	12	0	0.00	0.0–24.74
	Gungu	4	0	0.00	0.0–49.99	7	0	0.00	0.0–36.16
Sud Kivu	Shabunda	38	2	5.37	1.49–17.64	0	0	N/T	-
Kasai-Oriental or *Lomami*	Mwene-Ditu	50	9	18.37	9.97–31.42	0	0	N/T	-
	Ngandajika	100	1	1.00	0.02–5.56	0	0	N/T	-
Katanga or *anganyika*	Moba	23	2	8.87	2.47–27.34	3	0	0.00	0.0–57.30

CI, confidence interval; N/T, not tested.

Mongala, North Ubangi, South Ubangi and Kwilu are low-altitude regions (100 m – 700 m) characterised by tropical and equatorial climates with annual rainfalls of 1600 mm – 2000 mm, 90% relative humidity and an average annual temperature of 31 °C. These four provinces are prone to flooding during seasonal rainfall. Lomami is situated at an altitude of 700 m – 1200 m with a rainfall of 1200 mm/year – 1800 mm/year, an annual average temperature of 22 °C and 78% relative humidity. South Kivu is a high-altitude, mountainous region characterised by dense rainforests and an equatorial climate with an average annual rainfall of 1800 mm, an average temperature of 18 °C and 80% relative humidity. Tanganyika is located on the shores of Lake Tanganyika on the western branch of the Great Rift Valley in the humid tropical climate zone. The average annual temperature is 22 °C with an average annual precipitation of 700 mm and 85 °C of relative humidity.

The samples were collected from 2013 to 2015 from randomly selected sheep and goats following owners’ consent and under the supervision of the Animal Health and Production Department (DPSA) and the Central Veterinary Laboratory, Kinshasa. The location, age and sex were recorded for each animal. Samples from animals less than 4 months of age were discarded to avoid maternal antibodies. All farms visited had variable herd size ranging from 2 to 50. Blood samples were transported to the laboratory (Central Veterinary Laboratory [CVL], Kinshasa) on ice packs (approximately 4 °C). Twenty-four hours following whole blood collection, sera were harvested and kept at -20 °C until further use.

Sera were screened for the presence of specific immunoglobulin G (IgG) antibodies to RVFV using the ID Screen^®^ RVF Competition Multi-species enzyme-linked immunosorbent assay (ELISA) kits (ID-Vet; Innovative Diagnostics, Grabels, France). The reported sensitivity (Se) and specificity (Sp) values for this kit are 98% and 100%, respectively (Kortekaas et al. 2013). All IgG-positive sera were screened for specific anti-RVFV IgM antibodies using the ID Screen^®^ RVF IgM capture (ID-Vet; Innovative Diagnostics, Grabels, France), per manufacturers’ instructions.

Viral RNA was extracted from goat sera using a PureLink^®^ RNA Mini Kit (Invitrogen, Waltham, MA, United States [US]) and following the manufacturer’s instructions. For the reverse transcription polymerase chain reaction (RT-PCR), primers RVF1 and RVF2 were used to amplify a target segment of 551 bp (base pairs) which encodes a portion of the G2 glycoprotein gene as described previously (Tshilenge et al. 2018).

A descriptive statistical analysis was performed. True seroprevalence and exact 95% confidence interval (95% CI) were computed using an epidemiological calculator available at http://epitools.ausvet.com.au/content.php?page=TruePrevalence. This method calculates the true prevalence from survey testing results using a test of known sensitivity and specificity (Rogan & Gladen 1978). Comparisons of true seroprevalence between groups as well as associations between seropositivity were made using a nonparametric Fisher test. The threshold of significance was set at a *p* value of 0.05. Data analyses were performed using Epi info 7 (CDC, Atlanta, GA, US).

### Ethical considerations

The study protocol was implemented with approval from the Direction of Agriculture, Central Veterinary Laboratory of Kinshasa/Animal Health and Production Department. Consent for blood sampling of herds was obtained from owners. Animals were bled using conventional protocols.

## Results and discussion

A total of 893 sera were tested for anti-RVFV-nucleoprotein IgG antibodies by competitive ELISA. The true seroprevalence in goats ranged from 0.0% (95% CI 0.0–6.55) to 23.81% (95% CI 12.03–41.76), while in sheep, the true seroprevalence ranged from 0.0% (95% CI 0.0–7.56) to 37.11% (95% CI 15.48–65.94) (Table 1).

The seroprevalence varied significantly between the provinces from which samples originated, indicating that environmental and/or geographical factors play a role in RVF epidemiology in the DRC. The highest seroprevalences [37.11% (95% CI 15.48–65.94) for sheep and 23.81% (95% CI 12.03–41.76) for goats] were recorded in the low-altitude region of Businga, Nord-Ubangi province, where average rainfall ranges from 1600 mm to 1800 mm with a relative humidity of around 90%. This humid environment would explain higher numbers of potential insect vectors for RVFV compared to the less humid climates of Tanganyika and South Kivu where a seroprevalence of < 10% in both sheep and goats was recorded. It is known that during heavy rains, the numbers of fresh water species mosquitoes increase and play an important role in amplifying RVF (Ikegami & Makino 2011). Therefore, the monitoring and/or control of vectors for RVFV in regions of the DRC with high humidity would be opportune.

Low true seroprevalence values of < 10% were detected in the low-lying humid regions in the provinces of Sud-Ubangi, Mongala and Kwilu, suggesting that climate and the presence of insect vectors may not be the only factors involved in RVFV transmission in the DRC. The territories sampled in Nord-Ubangi province are located close to the border with the Central African Republic which has reported the presence of RVFV in the past (Gonzalez et al. 1989; Nakounne, Selekon & Morvan 2000), and so the transboundary movement of previously infected animals should also be taken into account when considering the results of this study.

The seroprevalences of > 10% recorded in Nord-Ubangi and Lomani are in agreement with seroprevalence values reported in other African countries, for example 24.7% in Madagascar (Jeanmaire et al. 2011), 29.7% and 22.0% in Tanzania (Sindato et al. 2015), 34% and 22% in Sudan (Eisa 1984), 85% and 12.2% in Mauritania and Cameroon (Zeller et al. 1995) and 19.3% in Senegal (Chevalier et al. 2005). In contrast, the seroprevalence values of < 10% recorded in Mongala, Kwilu, Sud Kivu and Tanganyika are similar to studies from Togo (i.e. 6.1%) (Zeller et al. 1995), Côte d’Ivoire (i.e. 9.71%) (Formenty, Domenech & Zeller 1992), Burkina Faso (i.e. 7.67 %) (Boussini et al. 2014) and Gabon (i.e. 6.47%) (Maganga et al. 2017).

A single sample from a goat was positive for IgM antibodies and the animal was confirmed by RT-PCR to be at the acute viraemic phase of RVF infection at the time of sampling. Amplification of RVFV nucleic acid from this serum by RT-PCR produced an expected DNA fragment of 551 bp. This confirms the utility of RT-PCR in the detection of early to acute viraemia which occurs between 4 and 8 days post-infection. The goat in question was identified in North Ubangi province which borders with the Central African Republic, once again highlighting the influence transboundary movement of previously infected animals can potentially have on the outcome of seroprevalence studies such as this one.

## Conclusion

Despite the lack of reports of RVF outbreaks in the DRC, it is evident that small ruminant populations in some parts of the country are exposed to the virus. More in-depth serological, molecular epidemiological and virological surveys of livestock need to be conducted in order to more fully understand the dynamics of RVFV circulation in the DRC. Vaccination in at-risk areas and the monitoring of vectors and animal movement should limit the spread of disease in susceptible animal populations and prevent transmission to humans.
